# Significance of induced hybridized metallic and non-metallic nanoparticles in single-phase nano liquid flow between permeable disks by analyzing shape factor

**DOI:** 10.1038/s41598-022-07251-y

**Published:** 2022-02-28

**Authors:** S. Bilal, Imtiaz Ali Shah, Muhammad Ramzan, Kottakkaran Sooppy Nisar, Ashraf Elfasakhany, Emad M. Eed, Hassan Ali S. Ghazwani

**Affiliations:** 1 Department of Mathematics, Air University, P.A.F Complex E-9, Islamabad, 44000 Pakistan; 2 Department of Computer Science, Bahria University, Islamabad, 44000 Pakistan; 3 Department of Mathematics, College of Arts and Sciences, Prince Sattam Bin Abdulaziz University, Wadi Aldawaser, 11991 Saudi Arabia; 4 Mechanical Engineering Department, College of Engineering, Taif University, P.O. Box 11099, Taif, 21944 Saudi Arabia; 5 Department of Clinical Laboratory Sciences, College of Applied Medical Sciences, Taif University, P.O.Box 11099, Taif, 21944 Saudi Arabia; 6 Department of Mechanical Engineering, Faculty of Engineering, Jazan University, Jazan, 45124 Kingdom of Saudi Arabia

**Keywords:** Software, Mechanical engineering

## Abstract

The current communication is designed by keeping in the mind high heat transfer capabilities of nanoliquids with the dispersion of diversified-natured nanoparticles in poorly conducting base liquids. Here, an amalgamation of metallic (Cu) and hybridization of metallic and non-metallic oxide (Cu-TiO_2_) nanoparticles to uplift thermophysical attributes of water is deliberated. The magnetically affected flow between rotating disks under the impact and permeability aspect is assumed. Empirical relations for effective dynamic viscosity, density, and heat capacitance to show mesmerizing features of obliged nanoparticles are also expressed. In addition, mathematical relations also depend on morphological factors like shape, size, and diameter of inducted nanoparticles. The mathematical formulation of the problem is conceded in the form of a system of ODEs after using similarity transformation on dimensional PDEs. Simulations of the complex coupled differential structure are solved by using a numerical approach by employing shooting and Runge–Kutta procedures jointly. The impact of flow concerning variables on associated distributions is revealed through tabular and graphical manner. Quantities of engineering interest associated with work like wall friction and thermal flux coefficients at walls of the disk are also calculated. It is deduced from an examination that the addition of metallic particles raises heat transfer more than non-metallic particles. A significant impression of magnetic field on shear stress is executed by hybrid nanoparticles along the surface of disks. In addition, elevation in Nusselt number and depreciation in skin friction coefficient is revealed against increasing magnitude of nanoparticle volume fraction. A positive trend in skin friction coefficient is manifested against the increasing magnitude of Reynold number. It is also observed that by increasing the size and shape of hybrid nanoparticles thermal conductivity and viscosity of the base fluid increases.

## Introduction

Liquids with enhanced heat transfer characteristics are utilized immensely in electronic and industrial devices. Water is considered a heat transferring liquid in past but due to low thermal conductivity, various approaches are adopted to enhance the thermophysical attributes and make it appropriate for use in multiple mechanical procedures. In this regards the most progressive approach found is the addition of nanoparticles by keeping in mind that suspension of solid particles raises the thermal conductivity of low conducting liquids. This concept was achieved after the milestone discovery of nanoparticles inaugurated by Choi^1^. Dispersion of these nanometric size particles in conventional liquids like water and oil leads to the production of an innovative class of liquids with improved thermal capabilities known as nanofluids. Nanoliquids are capitalized as automobile coolants, welding equipment, microwave tubes, laser diode array, solar paneling, refrigeration, thermal storage, boiler exhaustion, nuclear reactor cooling, and so forth. To make more effective use of nanofluids in different technological and industrial frameworks nanoparticles are characterized into various types on basis of structural variations, shapes, and chemical features such as metallic, non-metallic, polymeric, and nanotubes. Among these various kinds, metallic and non-metallic nanoparticles have owned much attention due to their unique structural and high aspect ratio features along with excellent thermal conductivities. Pandey et al.^2^ adumbrated the influence of different metallic oxide nanoparticles on hybridized stagnant single-phase flow over flat surfaces by incorporating Tiwari and Das model. Malavandi and Ganji^3^ investigated the migration of alumina nanoparticles inserted in water flowing in a channel under the impact of a magnetic field along with an analysis of convective heat transfer features. Haq et al.^4^ outlined magnetically influenced squeeze flow of nanoliquid over sensor surface by interacting base liquid homogenous mixture with copper, alumina, and titanium dioxide. Tahir et al.^5^ explicated advancement in the heat transfer rate of kerosene oil by adding magnetite ferrite particles with the placement of magnetized dipole. Vajravelu et al.^6^ conducted a study about the impression of nanoparticle volume fraction of silver and copper nanoparticles in producing elevated thermal aspects in water. Tassadiq et al.^7^ explored Von Karman's traditional flow over an infinite impermeable rotating disk with carrier fluid as water by induction of magnetized Ferro nanoparticles. Elgazery^8^ manifested magnetically influenced 2D nanoliquid flow over a porous stretching surface with the induction of silver, copper, alumina, and titania nanoparticles. Moghadassi et al.^9^ executed a computational approach to deliberate forced convective heat transfer in nanofluid flow over horizontally placed circular tube with induction of $\text{Al}_{2}{\text{O}}_{3}$ and $\text{Al}_{2} {\text{O}}_{3}{-}\text{Cu}$ hybrid nanoliquid.

Analysis of shape, size, aspect ratio and sphericity of nanoparticles is known as morphometry. It is a well-known fact that variation in morphological characteristics of nanoparticles definitely improves the features of hosting liquid. In addition, morphometric analysis of inducted particles plays a significant role in the use of differently structured nanoparticles in various emerging technological processes, optical fibers and biosensing, drug delivery system, biomolecular imaging, and bioanalytics. Afolalu et al.^10^ present a characterization of nanoparticles on basis of physio-chemical structuring and describe the capitalization of differently shaped particles in multidisciplinary fields. Haryadi et al.^11^ manifested the importance of the sphericity of nanoparticles and disclosed its role in biological phenomena by constructing a comparison with non-spherical nanoparticles. Albenese et al.^12^ revealed thermophysical relation correlating features of size, shape, surface charge, and chemical functionality and interpreted the change in flow attributes of nanofluid flow under mentioned attributes. Bhattad and Sarkar^13^ probed the performance of thermohydraulic evaporators by analyzing morphometric characteristics of hybridized particles. Sarma and Pandit^14^ deliberated free convective analysis of nanoliquid flow over an accelerated surface by using multi-shaped nanoparticles. Zeeshan et al.^15^ inserted different shaped copper nanoparticles to study fluid flow over swirling disk and measured irreversibility. Kashiie et al.^16^ adumbrated enhancement in thermal attributes of viscous Nanofluid flow between two parallel plates by adding Cu-Al_2_O_3_ hybrid particles under the implementation of the magnetic field in a transverse direction. They concluded that the heat transfer rate rises in the presence of the magnetic field. Khashiie et al.^17^ developed a non-uniqueness of solutions for the unsteady flow of hybrid Cu-Al_2_O_3_/water Nanofluid over a permeable stretching/shrinking disk. Wahid et al.^18^ carried out a study on Marangoni hybrid nanofluid flow over a permeable infinite disk immersed in a rotating disk. They adhered that volume fraction, porosity, and suction parameter enable the heat transfer rate to enhance and decelerate the boundary layer bifurcation. Some latest literature survey regarding enhancement in thermophysical features of nanoliquids by incorporating morphology is accumulated in^19^.

Nanofluids contain exclusive features that have attracted experts towards the development of new thermal engineering units. In the past mono nanofluids containing a single kind of particles are used to obtain certain benefits. However, some discrepancies in results regarding elevated heat transfer in liquids with insertion of nanoparticles is recorded in recent years. Due to this fact researchers have made efforts to the generation of new nanofluids by fusion of non-similar nanoparticles in a composite way known as hybridized nanofluid. This idea has produced better thermal attributes liquids instead of individual suspension. In this regards several promising investigations regarding hybridized nanofluids is commenced in recent years. Like, in 2017 Ranga et al.^20^ presented state of art analysis on the thermal capabilities of hybridized nanoparticles by constructing a comparison with the suspension of mono-natured nanoparticles. A review on enhancement in thermal characteristics of water base liquid by inserting hybrid was studied by Minea^21^. Zhou et al.^22^ probed minimization in an aggregation of nanoliquid with the addition of hybridized nanoparticles instead of insertion of ordinary surfactants. Kwak^23^ measured rheological characteristics of traditional poorly conducting liquid (ethylene glycol) by adding nanometer-sized particles of aspect ratio 3 and length of 10–30 nm and varying morphological aspects of embedded particles. Kabeel et al.^24^ conducted an experimental test regarding the performance of thermal exchangers by inducing alumina hybrid nanoparticles. Septiadi^25^ executed the importance of hybridized nanoparticles in the reduction of heavy-duty automobile engine coolant. Experimentation about the insertion of cooper-titania hybridized nanocomposites as the coolant in thermal exchanger was depicted by Madhesh and Kalaieselvam^26^. Khashiie et al.^27^ elucidated proficiency of induction of hybrid nanoparticles in elevation of the thermal characteristic of water under appliance of the magnetic field. They considered the combination of metal (Cu) and metal oxide $(\text{Al}_{2} \text{O}_{3})$ nanoparticles along with a depiction of tremendous augmentation in a heat transfer rate of base liquid in comparison to the addition of separate metallic and nonmetallic oxide particles. In recent years overwhelming studies related to the synthesis of organic and inorganic nanocarriers in ordinary liquids are done which can be accessed through^28–30^.

Magnetohydrodynamics is the study of flow dynamics under the interaction of magnetic fields. The term MHD was introduced by Alfeven et al.^31^. Magnetically influenced flows are valuable in numerous procedures and devices like MHD electricity generators, insulation of nuclear plants, crystallization, and so forth. Chamkha et al.^32^ demonstrated analytical and numerical methods for MHD flowing or thermal expansion in hybridized nanofluid via a porous stretching/shrinking layer with suction/injection and convective limiting conditions. Magnetized micropolar fluid flow past permeable cylinder was presented by Khader et al.^33^. Ijaz et al.^34^ reconnoitered magnetized hybrid nanofluid flow over stretchable spinning disks. Hayat et al.^35^ explored the magnetized movement of viscous fluid between two spinning disks. Ahmad et al.^36^ anticipated the flow of nanoliquid through a bi-directionally placed exponential stretching sheet under magnetic field influence. Usman et al.^37^ analyzed heat transfer aspects in the magnetized viscous liquid flowing over a non-linearly surface. Dogonchi et al.^38^ analyzed the impact of magnetically effected squeezing flow across parallel disks. Khader^33^ attained a solution of multi-walled nanotubes by implementing an analytical approach. Hosseinzadeh et al.^39^ probed anatomy aspects of hybridized nanofluid flow generated by spinning disks through a numerical scheme. Kashiie et al.^40^ analyzed the influence of different shape factors of (Cu-Al_2_O_3_) hybrid nanoparticles in water flow over the Riga surface by considering the aspects of radiative energy and EMHD. They incorporated three different shapes of hybrid nanoparticles (spherical, Birch, Blade) and found that nanoparticles concentration using blade shape is maximum while the spherical shape produces the lowest thermal rate. Recent studies on fluid flow problems under influence of magnetic fields are accessed through^39,41–48^.

From an overview of available literature, a very sparse research material related to the hybridization of metallic and metallic oxides along with consideration of shape and size factor of induced particles. So, the current work aims to analyze elevation in thermophysical characteristics of base liquid (water) with induction of metallic (Cu) and hybridization of metallic and non-metallic oxide (Cu-TiO_2_). We have considered flow between two coaxially rotated disks and the magnetic field is applied in the transverse direction to flow along with consideration of permeability aspects. Four different shapes of nanoparticles namely spherical, plates, bricks, and cylindrical are taken into account, and variation in shape and size factors are involved. To the best of the authors' knowledge, no such work is still done regarding the measurement of flow and heat characteristics of base liquids with insertion of metallic and non-metallic oxides along with analyzing the influence of shape and size. So, the authors have hoped that this work will provide direction to researchers working in this direction and will manipulate results by carrying morphological factors of induced particles. For the applications prospect of this work, we have considered unsteady, laminar, and 2D hybrid nanofluid flow and modeled governing equations in the form of ordinary differential equations after implementing the transformation approach. The shooting technique is applied to solve the constructed coupled differential system and the solution is analyzed against involved physical parameters by sketching associated profiles. Quantities of engineering interest like skin friction and Nusselt number are also computed. Validation of present findings by developing an agreement with previously published results is also presented.

## Mathematical formulation

**Figure 1:**
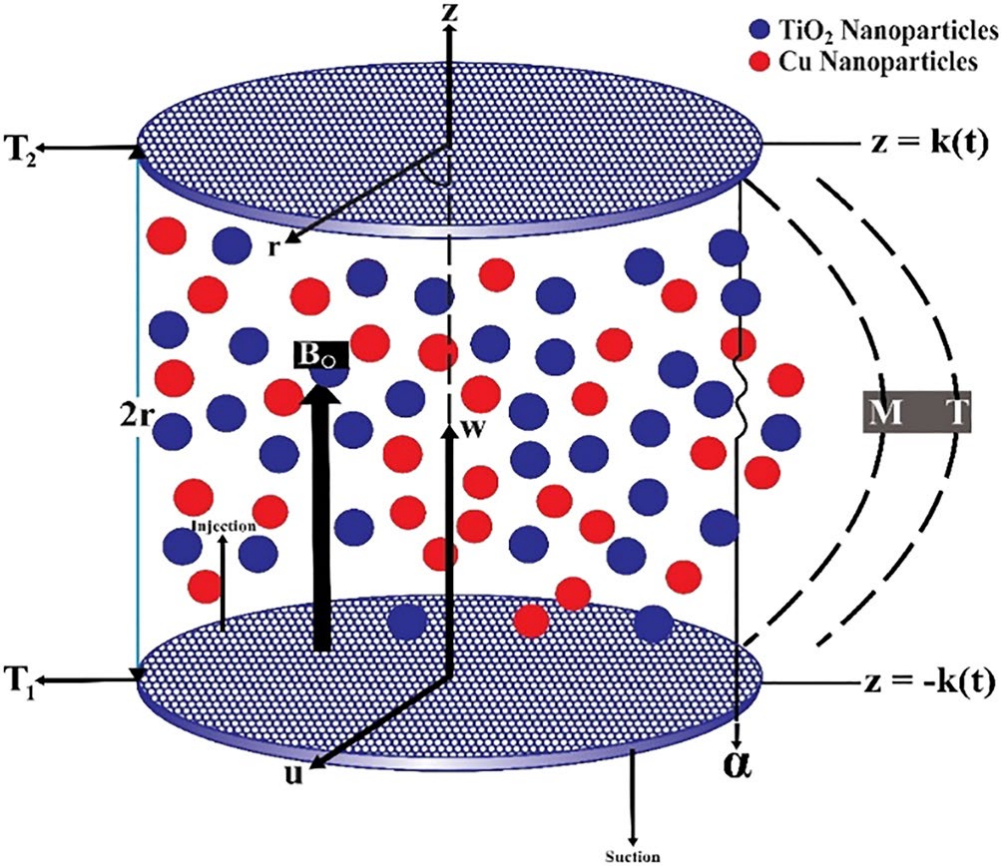
Physical model.

We have assumed 2D, non-turbulent, and time-dependent magnetized viscous liquid flowing among two parallel locating disks parted by separation $2k(t)$. While the lower disk lies at $-k(t)$ and the upper disk lies at the position of $k(t)$ on the other hand, the flow occurs due to convective conditions at porous surfaces. Hybrid nanoparticles constituted of titanium Oxides TiO_2_ and copper $(\text{Cu})$ are induced. The surface of disks considered to be permeable and under low magnetic Reynold number, the induced magnetic field must have vanished. The geometry of physical configuration is considered in the cylindrical coordinate system $(r,\theta ,z)$. Lower wall prescribed with temperature ${(T}_{1})$, where ${(T}_{2})$ is the temperature at the upper disk as displayed in Fig. 1.

The governing continuity, momentum, and energy equations for the present flow problem referred to^49^ are as under:

1 $$\frac{\partial u}{\partial r}+\frac{u}{r}+\frac{\partial w}{\partial z}=0,$$

2 $$\frac{\partial u}{\partial t}+\mathrm{u}\frac{\partial u}{\partial r}+\mathrm{w}\frac{\partial u}{\partial z}=-\frac{1}{{\rho }_{hnf}}\frac{\partial p}{\partial r}+{\upsilon }_{hnf} \left (\frac{{\partial }^{2}u}{\partial {r}^{2}}+\frac{1}{r}\frac{\partial u}{\partial r}-\frac{u}{{r}^{2}}+\frac{{\partial }^{2}u}{\partial {z}^{2}}\right )-\frac{{\sigma }_{e}{B}_{0}^{2}}{{\rho }_{hnf}}u,$$

3 $$\frac{\partial w}{\partial t}+\mathrm{u}\frac{\partial w}{\partial r}+\mathrm{w}\frac{\partial w}{\partial z}=-\frac{1}{{\rho }_{hnf}}\frac{\partial p}{\partial z}+{\upsilon }_{hnf}\left(\frac{{\partial }^{2}w}{\partial {r}^{2}}+\frac{1}{r}\frac{\partial w}{\partial r}+\frac{{\partial }^{2}w}{\partial {z}^{2}}\right),$$

4 $$\frac{\partial T}{\partial t}+\mathrm{u}\frac{\partial T}{\partial r}+\mathrm{w}\frac{\partial T}{\partial z}={\alpha }_{hnf}\frac{{\partial }^{2}T}{\partial {z}^{2}}.$$

where, $${\rho }_{hnf}$$ is the density of ($${HN}_{fd}$$), $${\sigma }_{e}$$ represents the electrical conductivity, $${B}_{0}^{2} $$ signifies the magnetic field strength, $${\alpha }_{hnf}$$ denotes thermal diffusivity.

### Applied models for thermophysical properties of $({{H}}{{{N}}}_{{{f}}{{d}}})$

The thermophysical properties of (${N}_{fd}$) convectional hybrid $({\text{TiO}}_{2}{-}\text{Cu}/\text{H}_{2}\text{O})$ then ${\varphi }_{1=}{\varphi }_{{TiO}_{2}}$, ${\varphi }_{2=}{\varphi }_{Cu}$, ${\rho }_{s1}={\rho }_{{TiO}_{2}}$, ${\rho }_{s2}={\rho }_{Cu}$, ${(\rho {c}_{p})}_{s1}={(\rho {c}_{p})}_{{TiO}_{2}}$, ${(\rho {c}_{p})}_{s2}={(\rho {c}_{p})}_{Cu}$, ${k}_{s1}={k}_{{TiO}_{2}}$ and ${k}_{s2}={k}_{Cu}$.

#### Effective dynamic viscosity

Efficient dynamic viscosity dependent on the standard (*HN*_*fd*_) ${\mu }_{hnf}$ and viscosity of (*HN*_*fd*_) ${\mu }_{hnf}$

$${\mu }_{hnf}=\frac{{\mu }_{bf}}{{(1-{\varphi }_{1}-{\varphi }_{2})}^{2.5}},$$

Abdelmalek et al.^51^ delivered a theory of diameter on viscosity:

5 $${\mu }_{hnf}={\mu }_{bf}\left(1+0.1008{ \left({ (\varphi }_{1}\right)}^{0.69574}{\left( {dp}_{1}\right)}^{0.44708}+{{ {(\varphi }_{2})}^{0.69574}\left( {dp}_{2}\right)}^{0.44708}) \right),$$

#### Effective density

The effective convectional (*HN*_*fd*_) density is denoted by the ${\rho }_{hnf}$:

6 $${\rho }_{hnf}={\varphi }_{1}{\rho }_{s1}+{\varphi }_{2}{\rho }_{s2}-\left(1-{\varphi }_{1}-{\varphi }_{2}\right){\rho }_{bf},$$

where, ${\rho }_{s1}$ or ${\rho }_{s2} \; and$ ${\rho }_{hnf}$ specify densities of solid NP’s and (*HN*_*fd*_).

#### Effective heat capacitance

The mathematical relation for heat capacitance is as under:

7 $${\rho }_{hnf}={\varphi }_{1}{(\rho {c}_{p})}_{s1}+{\varphi }_{2}({\rho {c}_{p})}_{s2}-\left(1-{\varphi }_{1}-{\varphi }_{2}\right){\left(\rho {c}_{p}\right)}_{bf},$$

where ${(\rho {c}_{p})}_{s1}$ and $({\rho {c}_{p})}_{s2}$ specify NP’s and (*B*_*fd*_) thermal capacitances.

#### Effective thermal conductivity

Thermal conductance of (*HN*_*fd*_) depending on specific size and shape factor of NP’s is described as:

8 $${k}_{hnf}=\frac{{k}_{s2}+{({n}_{2}-1)k}_{mbf}-({n}_{2}-1){\varphi }_{2}({k}_{mbf}-{k}_{s2})}{{k}_{s2}+({n}_{2}-1){km}_{bf}+{\varphi }_{2}({km}_{bf}-{k}_{s2})}{k}_{mbf}$$

where

$${k}_{mbf}=\frac{{k}_{s1}+{({n}_{1}-1)k}_{f}-({n}_{1}-1){\varphi }_{1}({k}_{f}-{k}_{s1})}{{k}_{s1}+({n}_{1}-1){k}_{f}+{\varphi }_{1}({k}_{f}-{k}_{s1})}{k}_{f}.$$

Here, ${k}_{hnf}$ is thermal conductivity for ${(HN}_{fd})$, ${k}_{s1}$ and ${k}_{s2}$ are thermal conductivities of solid NP’s, ${n}_{1}$ *and* ${n}_{2}$ are different size factors (spherical, bricks, plates, cylindrical), ${k}_{bf}$ and ${k}_{mbf}$ are thermal conductances for (*B*_*fd*_) and shape factor (*B*_*fd*_). The copper NP’s thermal conductivity is high but titanium oxide has low thermal conductivity in (*HN*_*fd*_). Table 1 represents the thermo-physical traits of the nanoparticles and the base fluid.

**Table 1:** Thermo-physical characteristics of (*HN*_*fd*_) and NPs referred to^49,50^.

Physical properties	${H}_{2}O \; (f)$	${TiO}_{2}\;({\varphi }_{1})$	Cu ${(\varphi }_{2}$)
$\rho$ (kg $\text{m}^{-3}$)	997.0	4250	8933
${C}_{p}$(J k $\text{g}^{-1}\,\text{k}^{-1})$	4180	686.2	385
$\kappa \; \left(\text{w} \, \text{m}^{-1} \, \text{k}^{-1}\right)$	0.6071	8.9538	400

Associated boundary conditions are as follows:

9 $$ \begin{gathered} z = - k(t)\;\;\;\;{\text{u}} = 0\;\;\;{\text{w}} = - A_{1} k^{\prime}(t)\;\;{\text{T}} = T_{1} , \hfill \\ z = k(t)\;\;\;\;{\text{u}} = 0\;\;\;{\text{w}} = - A_{1} k^{\prime}(t)\;\;{\text{T}} = T_{2} . \hfill \\ \end{gathered} $$

Here, ${A}_{1}$ is the metric of penetrability of the partition. Following variables are suggested after regulating the equation and removing the pressure term.

10 $$\eta =\frac{z}{k} \;\;\; \mathrm{u}=-\frac{r{\upsilon }_{f}}{{k}^{2}}{F}_{\eta }\left(\eta ,t\right)\;\;\;  \mathrm{w}=\frac{2{\upsilon }_{f}}{k}F\left(\eta ,t\right) \;\;\; \theta =\frac{T-{T}_{2}}{{T}_{1}-{T}_{2}},$$

and result in

11 $$\frac{{\upsilon }_{hnf}}{{\upsilon }_{f}}{F}_{\eta \eta \eta \eta }+\alpha \left(3{F}_{\eta \eta }+\eta {F}_{\eta \eta \eta }\right)-2F{F}_{\eta \eta \eta }-\frac{{k}^{2}}{{\upsilon }_{f}}{F}_{\eta \eta t}-\frac{{\rho }_{f}}{{\rho }_{hnf}}\mathrm{M}{F}_{\eta \eta }=0,$$

12 $$\frac{{\upsilon }_{hnf}}{{\upsilon }_{f}}{F}_{\eta \eta \eta \eta }+\alpha \left(3{F}_{\eta \eta }+\eta {F}_{\eta \eta \eta }\right)-2F{F}_{\eta \eta \eta }-\frac{{k}^{2}}{{\upsilon }_{f}}{F}_{\eta \eta t}-\frac{{\rho }_{f}}{{\rho }_{hnf}}\mathrm{M}{F}_{\eta \eta }=0,$$

13 $${\theta }_{\eta \eta }+\frac{{\upsilon }_{f}}{{\alpha }_{hnf}}(\alpha \eta -2F){\theta }_{\eta }-\frac{{k}^{2}}{{\alpha }_{hnf}}{\theta }_{t}=0.$$

With Bc’s

14 $$\eta =-1, \; F=-R, {F}_{\eta }=0, \; \theta =1, \; \mathrm{ and} \; \eta =1, \; F=R, \; {F}_{\eta }=0, \; \theta =0.$$

Here, α = $\frac{k{k}^{{\prime}}\left(t\right)}{{\upsilon }_{f}}$ is wall expansion ratio, *R* = $\frac{{A}_{1}k{k}^{{\prime}}\left(t\right)}{2{\upsilon }_{f}}$ is the absorptivity Reynolds number and M = $\frac{{\sigma }_{e}{B}_{0}^{2}{k}^{2}}{{\mu }_{f}}$ is the magnetic parameter. Finally, we set F = $f$ *R*, α is a constant, $f$ = $f$ (η) and θ = θ (η), which leads to ${\theta }_{t}$ = 0 and ${f}_{\eta \eta t}$ = 0.

Thus, we attained the following form:

15 $$\frac{{\upsilon }_{hnf}}{{\upsilon }_{f}}{f}_{\eta \eta \eta \eta }+\alpha \left(3{f}_{\eta \eta }+\eta {f}_{\eta \eta \eta }\right)-2Rf{f}_{\eta \eta \eta }-\frac{{\rho }_{f}}{{\rho }_{hnf}}M{f}_{\eta \eta }=0,$$

16 $${\theta }_{\eta \eta }+\left(\left(1-({\varphi }_{1}+{\varphi }_{2})\right)+({\varphi }_{1})\left(\frac{{\rho }_{{cps}_{1}}}{{\rho }_{cpbf}}\right)+({\varphi }_{2})\left(\frac{{\rho }_{cp{s}_{2}}}{{\rho }_{cpbf}}\right)\right)\frac{ {k}_{mbf} }{ {k}_{hnf} }\frac{ {k}_{bf} }{ {k}_{mbf} }\mathrm{Pr}\left(\alpha \eta -2Rf\right){\theta }_{\eta }=0$$

17 $$ \upeta  = - {1}; \; f =  - {1}, \; f_{\eta } = 0, \; \theta  = { 1}, \; {\text{ and}} \; \upeta  = 1; \; f = { 1}, \; f_{\eta } =  0, \;  \theta  =  0. $$

where, $${\upsilon }_{hnf}=\frac{{\mu }_{hnf}}{{\rho }_{hnf}}$$, $${\alpha }_{hnf}=\frac{{k}_{hnf}}{{{(\rho c}_{p})}_{hnf} } \; and \; Pr=\frac{{(\mu {C}_{p})}_{bf}}{{k}_{bf}}$$.

After expressing empirical relation of hybrid nanofluid in Eqs. (15)–(16), the following mathematical equations are found:

18 $$\left(\frac{1}{{\left(1-({\varphi }_{1}+{\varphi }_{2})\right)}^{2.5}\left(\left(1-({\varphi }_{1}+{\varphi }_{2})\right)+{(\varphi }_{1})\left(\frac{{\rho }_{{s}_{1}}}{{\rho }_{bf}}\right)+({\varphi }_{2})\left(\frac{{\rho }_{{s}_{2}}}{{\rho }_{bf}}\right)\right)}\right){f}^{{{\prime}}{{\prime}}{{\prime}}{{\prime}}}\left[\eta \right]+\alpha \left(3{f}^{{^{\prime}}{^{\prime}}}\left[\eta \right]+\eta {f}^{{{\prime}}{{\prime}}{{\prime}}}\left[\eta \right]\right)-2Rf\left[\eta \right]{f}^{{{\prime}}{^{\prime}}{^{\prime}}}\left[\eta \right]-\left(\frac{1}{\left(\left(1-{\varphi }_{1}-{\varphi }_{2}\right)+{\varphi }_{1}\left(\frac{{\rho }_{{s}_{1}}}{{\rho }_{bf}}\right)+{\varphi }_{2}\left(\frac{{\rho }_{{s}_{2}}}{{\rho }_{bf}}\right)\right)}\right)\mathrm{M} {f}^{{{\prime}}{{\prime}}}\left[\eta \right]=0,$$

19 $${\theta }^{{{\prime}}{{\prime}}}\left[\eta \right]+\left(\left(1-\left({\varphi }_{1}+{\varphi }_{2}\right)\right)+\left({\varphi }_{1}\right)\left(\frac{{\rho }_{{cps}_{1}}}{{\rho }_{cpbf}}\right)+\left({\varphi }_{2}\right)\left(\frac{{\rho }_{cp{s}_{2}}}{{\rho }_{cpbf}}\right)\right)\left(\frac{{k}_{s2}+\left(N-1\right){k}_{mbf}+{\varphi }_{2}\left({k}_{mbf}-{k}_{s2}\right)}{{k}_{s2}+{\left(N-1\right)k}_{mbf}-\left(N-1\right){\varphi }_{2}\left({k}_{mbf}-{k}_{s2}\right)}\right)\left(\frac{{k}_{s1}+\left(N-1\right){k}_{bf}+{\varphi }_{1}\left({k}_{bf}-{k}_{s1}\right)}{{k}_{s1}+{\left(N-1\right)k}_{bf}-\left(N-1\right){\varphi }_{1}\left({k}_{bf}-{k}_{s1}\right)}\right) Pr\left(\alpha \eta -2Rf\left[\eta \right]\right){\theta }^{{\prime}}\left[\eta \right]=0$$

Empirical relations in the form of coefficients are expressed as under:

20 $${H}_{1}=\left(\frac{1}{{\left(1-({\varphi }_{1}+{\varphi }_{2})\right)}^{2.5}\left(\left(1-({\varphi }_{1}+{\varphi }_{2})\right)+({\varphi }_{1})\left(\frac{{\rho }_{{s}_{1}}}{{\rho }_{bf}}\right)+({\varphi }_{2})\left(\frac{{\rho }_{{s}_{2}}}{{\rho }_{bf}}\right)\right)}\right),$$

21 $${H}_{2}=\left(\frac{1}{\left(\left(1-{\varphi }_{1}-{\varphi }_{2}\right)+{\varphi }_{1}\left(\frac{{\rho }_{{s}_{1}}}{{\rho }_{bf}}\right)+{\varphi }_{2}\left(\frac{{\rho }_{{s}_{2}}}{{\rho }_{bf}}\right)\right)}\right),$$

22 $${H}_{3}=\left(\left(1-({\varphi }_{1}+{\varphi }_{2})\right)+({\varphi }_{1})\left(\frac{{\rho }_{{cps}_{1}}}{{\rho }_{cpbf}}\right)+{(\varphi }_{2})\left(\frac{{\rho }_{cp{s}_{2}}}{{\rho }_{cpbf}}\right)\right),$$

23 $${D}_{1}=\left(\frac{{k}_{s2}+\left(N-1\right){k}_{mbf}+{\varphi }_{2}\left({k}_{mbf}-{k}_{s2}\right)}{{k}_{s2}+{\left(N-1\right)k}_{mbf}-\left(N-1\right){\varphi }_{2}\left({k}_{mbf}-{k}_{s2}\right)}\right),$$

24 $${D}_{2}=\left(\frac{{k}_{s1}+\left(N-1\right){k}_{bf}+{\varphi }_{1}\left({k}_{bf}-{k}_{s1}\right)}{{k}_{s1}+{\left(N-1\right)k}_{bf}-\left(N-1\right){\varphi }_{1}\left({k}_{bf}-{k}_{s1}\right)}\right),$$

25 $$\omega ={D}_{1}{D}_{2},$$

Putting values of (21)–(25) in Eqs. (18) and (19), the following equations are formed:

26 $${H}_{1}f{^{\prime}}{^{\prime}}{^{\prime}}{^{\prime}}[\eta ]-\alpha (3{f}^{{{\prime}}{{\prime}}}\left[\eta \right]+\eta f{^{\prime}}{^{\prime}}{^{\prime}}[\eta ])-2Rf[\eta ]f{^{\prime}}{^{\prime}}[\eta ]-{H}_{2}\mathrm{M}f{^{\prime}}{^{\prime}}[\eta ]=0,$$

27 $$\theta {^{\prime}}{^{\prime}}[\eta ]+{H}_{3}\omega \mathrm{Pr}(\alpha \eta -2Rf\left[\eta \right])\theta {^{\prime}}[\eta ]=0.$$

Boundary condition

28 $$ {\text{at }} \; \upeta = - {1}; \; f =  - {1}, \; f_{\eta } =  0, \;  \theta  =1 \; {\text{ and at }} \; \eta  = { 1}; \; f = { 1}, \; f_{\eta } =  0, \; \theta  =  0. $$

### Quantities of engineering interest

#### Wall drag coefficients

The wall drag coefficients at surfaces of disks ${C}_{f1}$ and ${C}_{f-1}$ respectively are illustrated as under

29 $${C}_{f-1}=\frac{{\xi }_{zr}{|}_{\upeta = -1}}{{\rho }_{f}{({k}^{{\prime}}{A}_{1})}^{2}}=\frac{1}{{Re}_{r}{(1-{\varphi }_{1}-{\varphi }_{2})}^{2.5}} {f}^{{{\prime}}{{\prime}}}\left(-1\right),$$

30 $${C}_{f1}=\frac{{\xi }_{zr}{|}_{\upeta = 1}}{{\rho }_{f}{({k}^{{\prime}}{A}_{1})}^{2}}=\frac{1}{{Re}_{r}{(1-{\varphi }_{1}-{\varphi }_{2})}^{2.5}}{f}^{{{\prime}}{{\prime}}}\left(1\right).$$

where, ${R}_{{\varvec{r}}}=4\left(\frac{k}{r}\right){(\frac{1}{{\varvec{R}}})}^{2}$ is the local Reynolds number.

31 $${\xi }_{zr}={\mu }_{hnf}\left(\frac{\partial u}{\partial z}\right){|}_{\upeta = -1}=\frac{{\mu }_{bf}}{{\left(1-{\varphi }_{1}-{\varphi }_{2}\right)}^{2.5}}\left(\frac{r{\upsilon }_{f}}{{k}^{3}}\right){f}^{{{\prime}}{{\prime}}}\left(-1\right),$$

32 $${\xi }_{zr}={\mu }_{hnf} \left(\frac{\partial u}{\partial z} \right){|}_{\upeta = 1}=\frac{{\mu }_{bf}}{{(1-{\varphi }_{1}-{\varphi }_{2})}^{2.5}}(\frac{r{\upsilon }_{f}}{{k}^{3}}){f}^{{{\prime}}{{\prime}}}\left(1\right),$$

#### Nusselt numbers

The (Nusselt numbers) at lower and upper walls ${Nu|}_{\upeta = -1}$ and ${Nu|}_{\upeta = 1}$ are described as under:

33 $${Nu|}_{\upeta = -1}=\frac{k{s}_{z}}{{\kappa }_{f}({T}_{1}-{T}_{2})}{|}_{\upeta = -1}=-\frac{{k}_{hnf}}{{k}_{f} }{\theta }^{{\prime}}\left(-1\right)$$

34 $${Nu|}_{\upeta = 1}=\frac{k{s}_{z}}{{\kappa }_{f}({T}_{1}-{T}_{2})}{|}_{\upeta = 1}-\frac{{k}_{hnf}}{{k}_{f} }{\theta }^{{\prime}}\left(1\right),$$

where heat flux is signified as ${s}_{z}$ which is follows as:

35 $${s}_{z}{|}_{\upeta = -1}=-{k}_{hnf} \left(\frac{\partial T}{\partial z}\right){|}_{\upeta = -1}=-\frac{({T}_{1}-{T}_{2})}{k}{k}_{hnf}{\theta }^{{\prime}}\left(-1\right)$$

36 $${s}_{z}{|}_{\upeta = 1}=-{k}_{hnf}\left(\frac{\partial T}{\partial z}\right){|}_{\upeta = 1}=-\frac{\left({T}_{1}-{T}_{2}\right)}{k}{k}_{hnf}{\theta }^{{\prime}}\left(1\right),$$

where, $$R=\frac{{A}_{1}k{k}^{{\prime}}\left(t\right)}{2{\upsilon }_{f}}$$.

### Numerical solution

This section discloses the basic steps involved in the implementation of combined numerical schemes known as shooting and RK schemes.

Firstly, we initialized with the introduction of the following variables:

37 $${q}_{1}^{*}=f\left[\eta \right], {q}_{2}^{*}={f}^{{\prime}}\left[\eta \right], {q}_{3}^{*}= {f}^{{{\prime}}{{\prime}}}\left[\eta \right],{q}_{4}^{*}= f{^{\prime}}{^{\prime}}{^{\prime}}[\eta ],{q}_{5 }^{*}= \theta \left[\eta \right], {q}_{6}^{*}=\theta {^{\prime}}[\eta ]$$

First, transform the model in the following pattern in equation No. (18)–(19):

38 $${f}^{{{\prime}}{{\prime}}{{\prime}}{{\prime}}}\left[\eta \right]=\frac{1}{{B}_{1}}({f}^{{{\prime}}{{\prime}}{{\prime}}}\left[\eta \right]\left(-\alpha \eta +Ref\left[\eta \right]\right)-{f}^{{{\prime}}{{\prime}}}\left[\eta \right]\left(3\alpha +Re{f}^{{\prime}}\left[\eta \right]\right)+2Re(\alpha {f}_{\eta \eta }+{B}_{2}\mathrm{M}{f}_{\eta \eta })$$

39 $${\theta }^{{{\prime}}{{\prime}}}\left[\eta \right]={B}_{3}\beta \mathrm{Pr}(-\alpha \eta +2Ref\left[\eta \right])\theta {^{\prime}}[\eta ].$$

After replacement of variables the following system is obtained:

40 $$\left[\begin{array}{c}{q}_{1}^{*{{\prime}}}\\ {q}_{2}^{*{{\prime}}}\\ {q}_{3}^{*{{\prime}}}\\ {q}_{4}^{*{{\prime}}}\\ {q}_{5}^{*}{{\prime}}\\ {q}_{6}^{*{{\prime}}}\end{array}\right]=\left[\begin{array}{c}{q}_{2}^{*}\\ {q}_{3}^{*}\\ {q}_{4}^{*}\\ \frac{1}{{B}_{1}}({f}^{{{\prime}}{{\prime}}{{\prime}}}\left[\eta \right]\left(-\alpha \eta +Ref\left[\eta \right]\right)-{f}^{{{\prime}}{{\prime}}}\left[\eta \right]\left(3\alpha +Re{f}^{{\prime}}\left[\eta \right]\right)+2{R}_{0}\alpha {f}_{\eta \eta }+{B}_{2}M{f}_{\eta \eta } )\\ {q}_{6}^{*}\\ {B}_{3}\beta Pr(-\alpha \eta +2Ref\left[\eta \right])\theta {^{\prime}}[\eta ]\end{array}\right] .$$

Consequently, the initial conditions are:

$$\left[\begin{array}{c}{q}_{1(\upeta = -1)}^{*}\\ {q}_{2(\upeta = -1)}^{*}\\ {q}_{3(\upeta = 1)}^{*}\\ {q}_{4(\upeta = 1)}^{*}\\ {q}_{5(\upeta =- 1)}^{*}\\ {q}_{6(\upeta = 1)}^{*}\end{array}\right]=\left[\begin{array}{c}-1\\ 0\\ a\\ b\\ 1\\ 0\end{array}\right],$$

where a and b missing initial conditions and are approximated by guessed values to achieve the required accuracy.

### Validation of results

#### Interpretation of results

This section is presented to explain the influence of involved physical variables like Reynold number $(R)$, expansion ratio $({\upalpha })$, Prandtl number $(Pr)$, Magnetic parameter $(M)$, nanoparticles volume fraction $(\varphi )$, ${(\varphi }_{1})$ and ${(\varphi }_{2})$ nanoparticle volume fractions of ($\text{TiO}_{2}),$ $(\text{Cu})$ respectively on axial and radial components of velocity along with temperature and concentration distributions.

**Figure 2:**
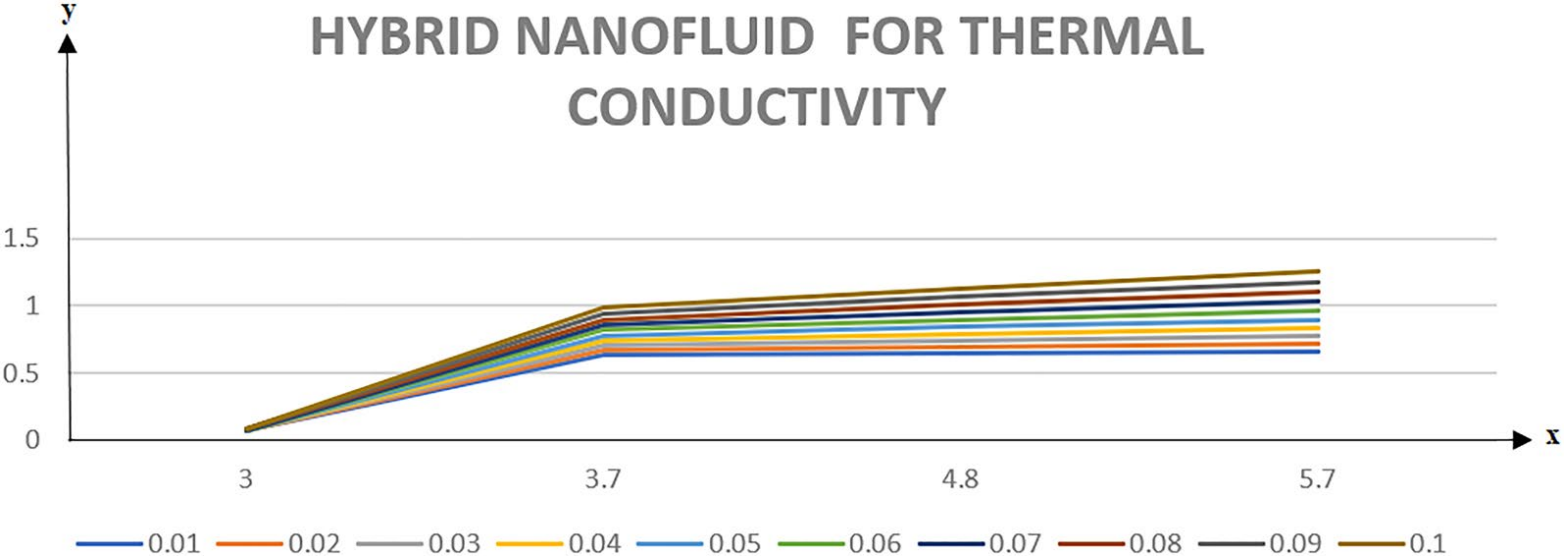
Predicted thermal conductivity variation for the (*HN*_*fd*_).

**Figure 3:**
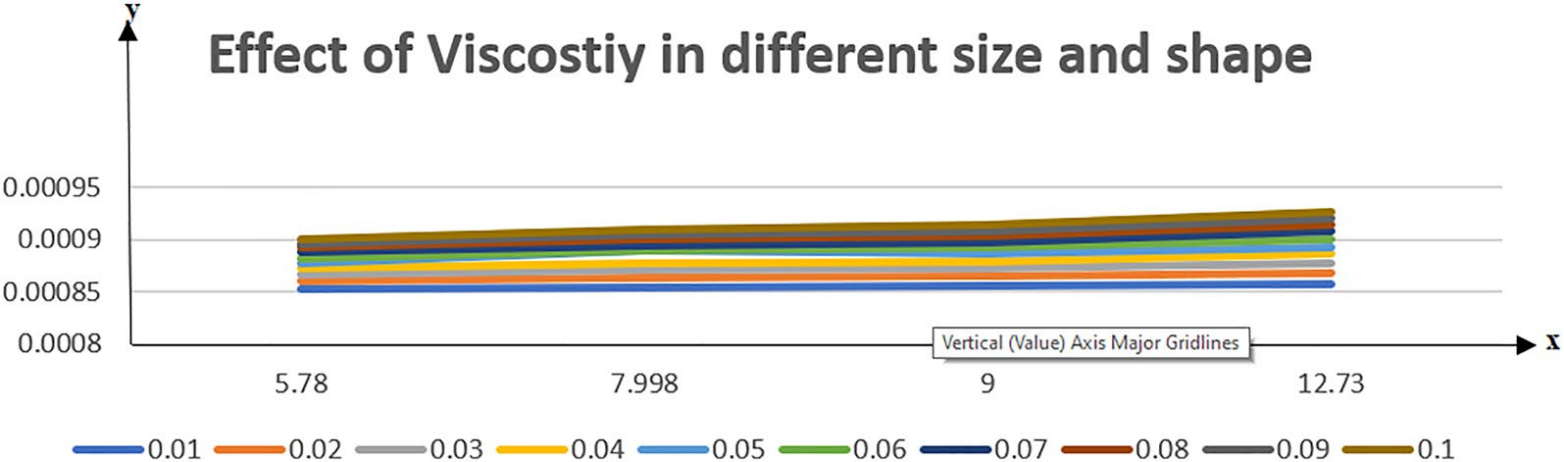
Variation in viscosity against different sizes and shapes of hybrid NPs.

**Figure 4:**
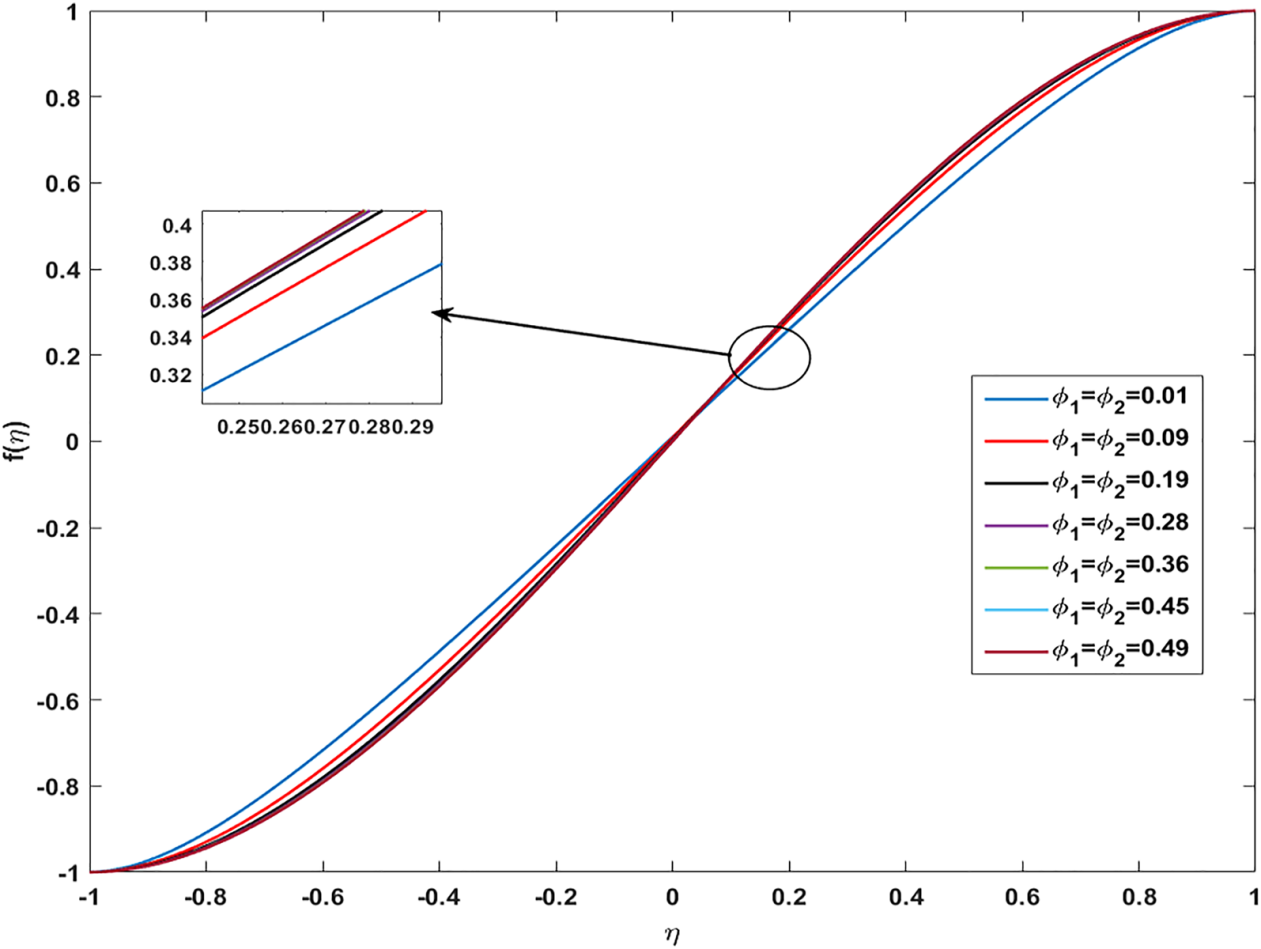
Variation in Axial velocity profile against volume fraction for $\alpha =-4, \, R=-1, \, M=1, \, Sc=1, \, Pr=6.2.$

**Figure 5:**
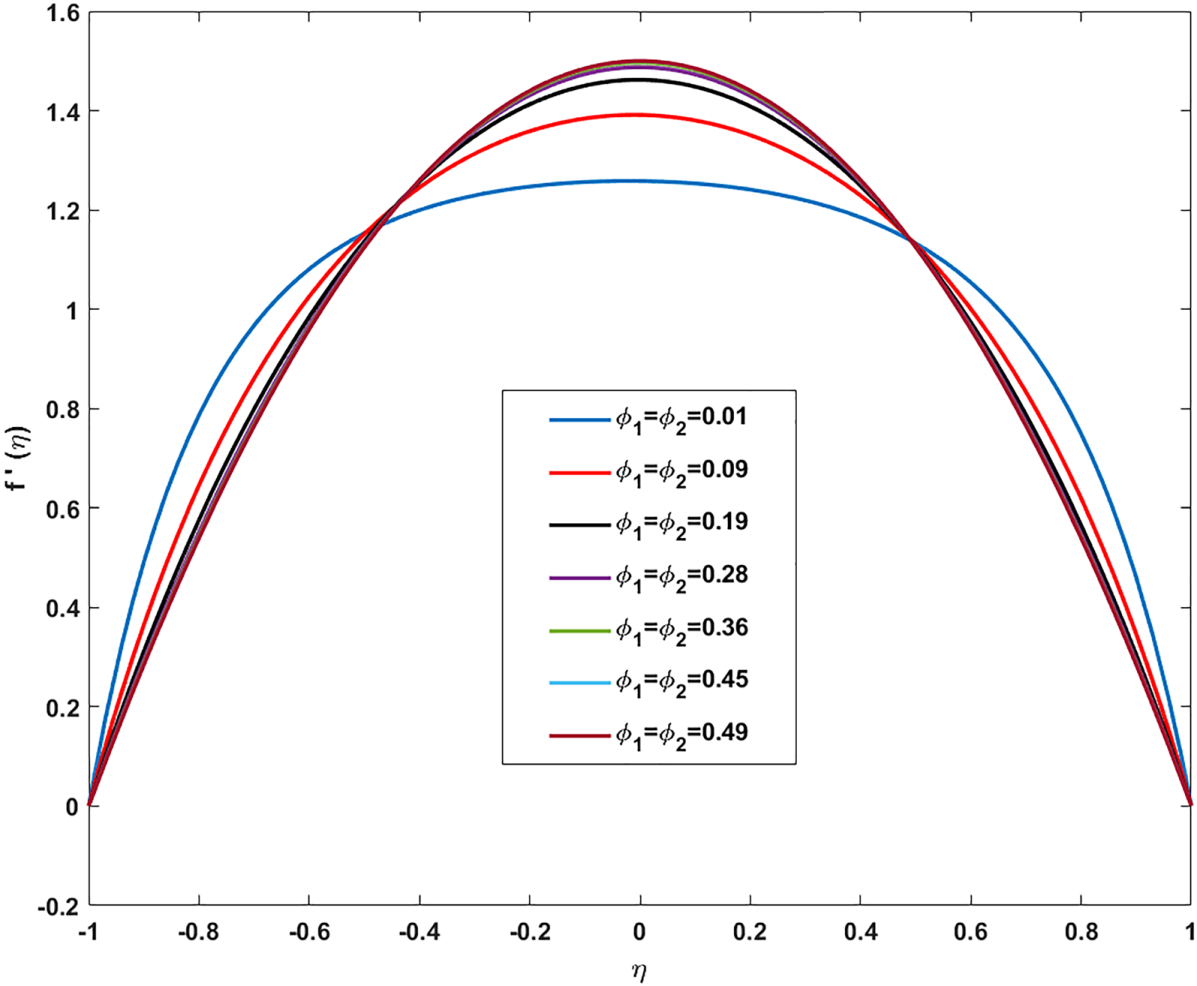
Variation in Radial velocity profile against volume fraction for $\alpha =-4, \, R=-1, \, M=1, \, Sc=1, \, Pr=6.2.$

**Figure 6:**
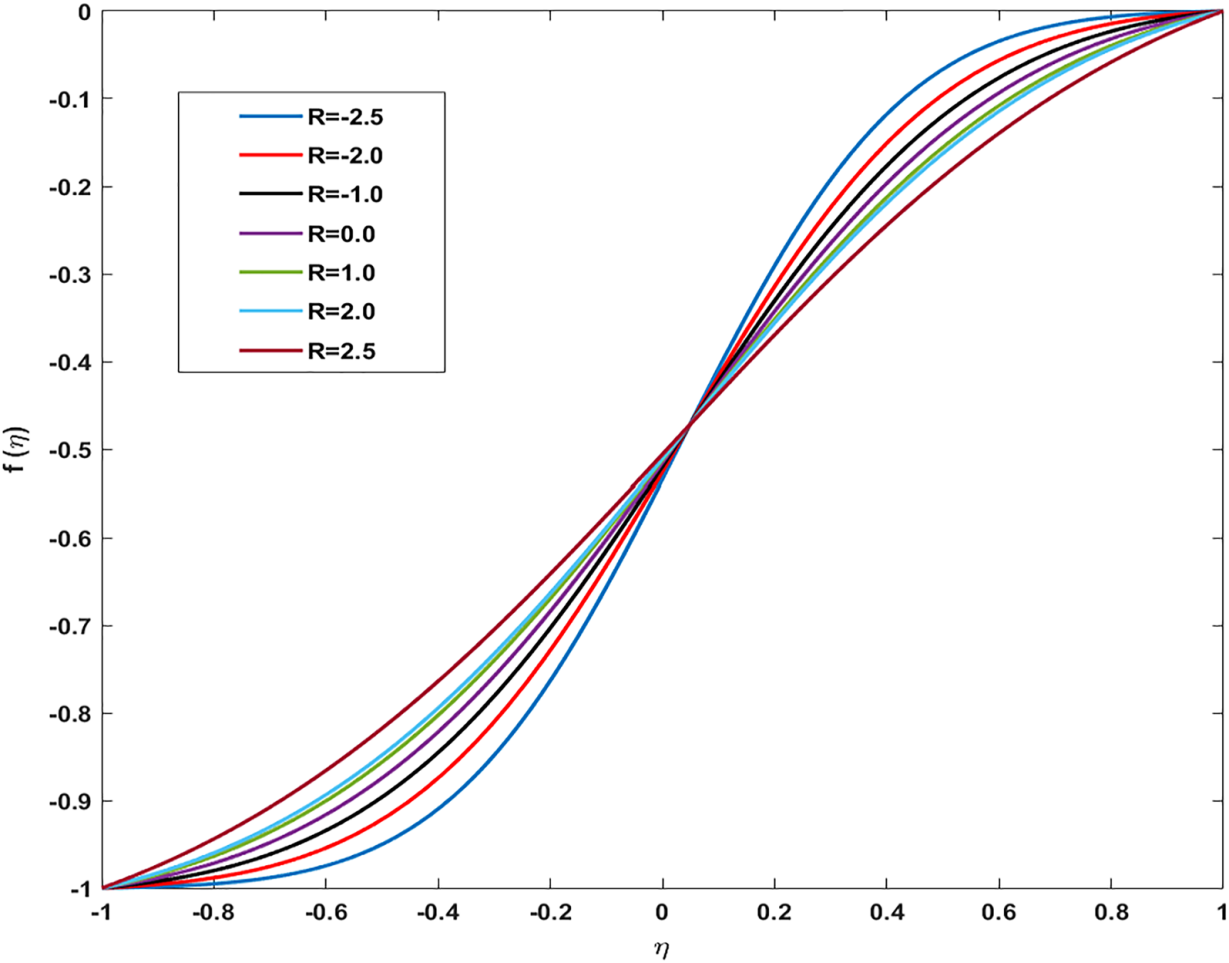
Variation in Axial velocity profile against Reynold number for $\alpha =5,{\varphi }_{1}={\varphi }_{2}=0.01, \, M=1, \, Sc=1, \, Pr=6.2$.

**Figure 7:**
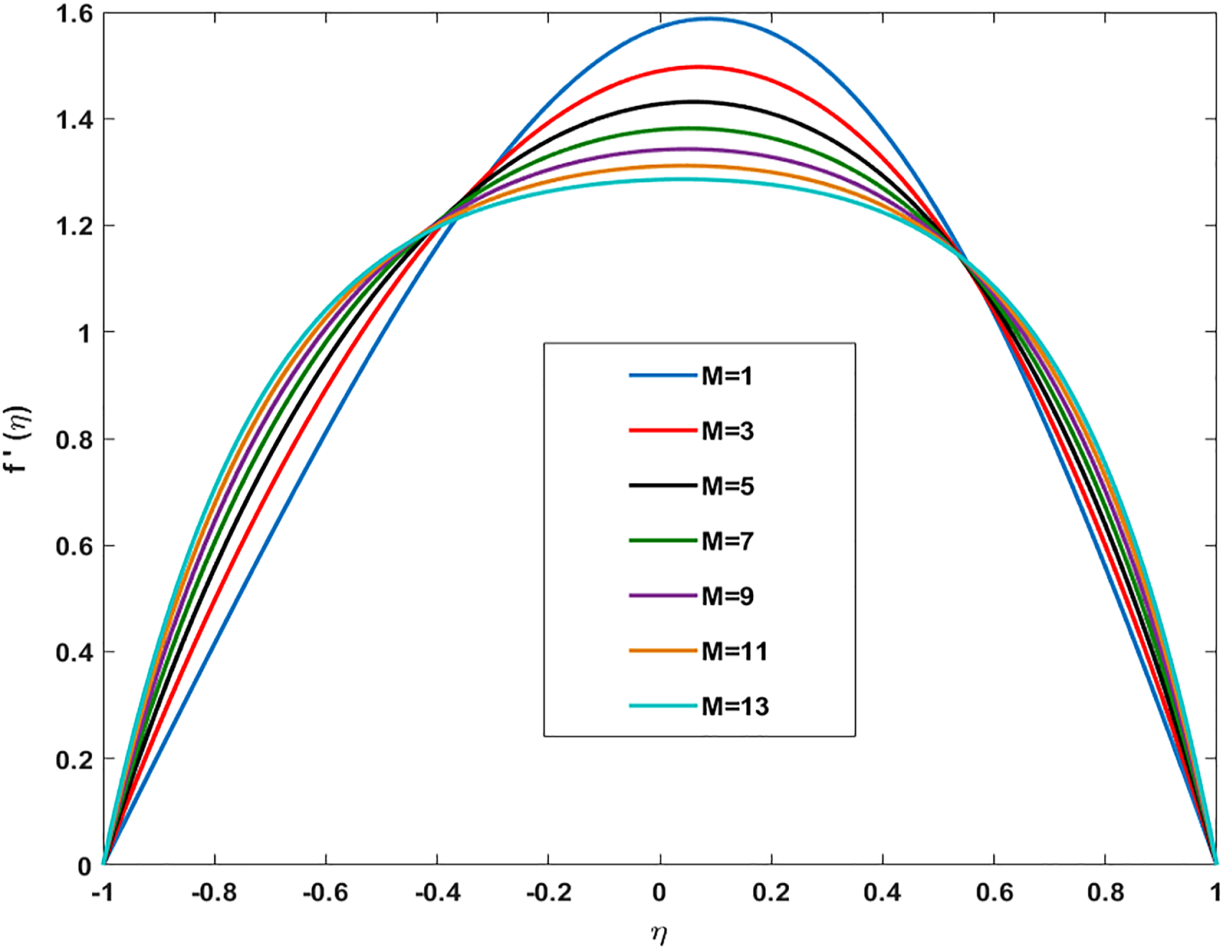
Variation in Radial velocity profile against magnetic parameter (M) for $\alpha =5,{\varphi }_{1}={\varphi }_{2}=0.01, \, M=1, \, Sc=1, \, Pr=6.2$.

**Figure 8:**
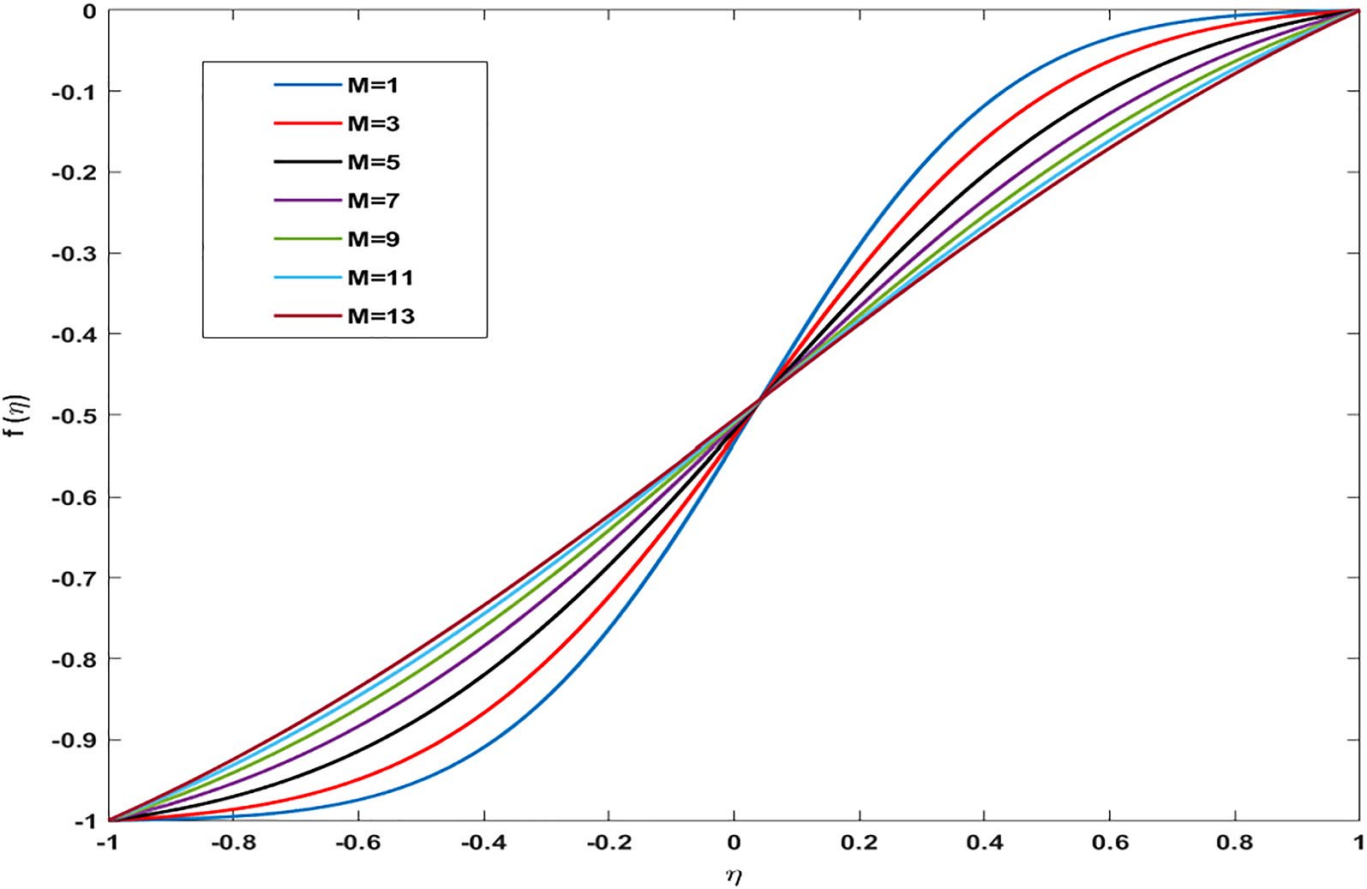
Variation in Axial velocity profile against magnetic parameter for $\alpha =4, \, R=-1, \, {\varphi }_{1}={\varphi }_{2}=0.04, \, Sc=1, \, Pr=6.2.$

Table 2 discloses the influence of nanoparticles volume fraction volume $$(\varphi )$$, Reynold number $$(R)$$ and expansion ratio parameter $$({\upalpha })$$ on skin friction and heat flux coefficient produced insertion of $$(\text{TiO}_{2}{-}\text{Cu})$$ hybridized nanoparticles in water at the surface of the lower disk. Four different shapes of induced nanoparticle spherical, plates, bricks, and cylindrical are considered and heat flux is also calculated. It is evaluated that by increasing $$(\varphi )$$ skin friction coefficient decreases whereas it mounts for increasing value of $$({\upalpha })$$ and $$(R)$$. It is because by increasing all these mentioned physical parameters viscosity of fluid decreases and fluid particles move with the excessive speed within the disk so less friction is offered to the fluid by surfaces of disks. In addition, it is justified by the fact that nanoparticle volume fraction enhances the thermal conductance, and thus as outcome average kinetic energy raises which lift the motion of fluid and causes a reduction in wall drag forces. Regarding variation in heat flux coefficient, it is observed that an increase in of $$({\upalpha })$$ and $$(R)$$ temperature gradient enhances whereas contrary behavior is depicted against $$({\upalpha })$$. It is worthwhile to notice that the high magnitude of heat flux at the wall is computed by inserting platelets shape $$({\text{TiO}}_{2}{-}\text{Cu})$$ hybridized particle. This is because Reynold number (R) is the ratio of inertial to viscous forces. So, by increasing (Re) influence of viscous forces decrements and kinetic motion of particles enhances. This boost in kinetic energy is the measure of the temperature so it enriches, and heat flux magnitude also raises. Table 3 illustrates the variation in skin friction and thermal flux coefficients at the lower wall by varying Reynold number $$(R)$$, and magnetic parameter ($$M)$$ along with restricting expansion ratio ($$\alpha $$) greater than zero, It is manifested that with increment in magnetic parameter skin friction coefficient enhances and Nusselt number also uplifts. It is because of the reason that by increasing magnetic parameter (M) magnitude of Lorentz forces generated due to transversal appliance of the magnetic field to fluid flow which ceases the motion of particles and creates a reduction in kinetic energy. Due to this impact, the magnitude of shear forces applied by the surface i.e. measured by skin friction coefficient evidently enhances. Table 4 shows the accuracy of the applied computational scheme by finding values of missing conditions at different values of $$\eta $$. From the attained data it is observed that convergence criteria meet for evaluation of results. Table 5 reveals the credibility of the present work by establishing a comparison with results published by Kashif et al.^49^. For the agreement of results currently computed problem is restricted by setting Reynold number $$(R=0)$$, Prandtl number $$(Pr=0)$$, Magnetic parameter $$(M=0)$$. Results are compared for skin friction and heat transfer coefficients by varying expansion ratio ($$\alpha $$) varying from expansion ratio ($$-5\le \alpha \le 5$$). An excellent match of results is found which shows the assurance and reliability of results. Variation in thermal conductivity of base fluid by taking into account the aspects of shape factor $$(n)$$ of induced nanoparticles is revealed in Fig. 2. Here, four different shapes of particles namely, spherical, plates, bricks, and cylindrical are obliged along with the assumption of consideration of the same magnitude of nanoparticle volume fractions i.e. $${\varphi }_{1}$$ = $${\varphi }_{2}$$ = $$\varphi $$. It is noticed from the sketch that by increasing the shape factor of particles thermal conductivity enhances and optimized magnitude is attained at $$n = 5.7$$ (platelets). It is also depicted that by increasing nanoparticle volume fraction ranging from $$(0.01\le \varphi \le 0.1)$$ elevation in thermal conductivity is manifested. Deviation in viscosity of base fluid (water) versus change in shape and size of inserted nanoparticles along with consideration of different magnitudes of particle volume fraction is portrayed in Fig. 3. Here, four different particles (spherical, bricks, cylindrical, platelets) are assigned by shape factor $$(n)$$ with magnitudes $$(5.78, 7.889, 9, 12.7)$$ respectively. It is checked that by elevating shape factor $$(n)$$ and particle volume fraction $$(\varphi )$$ viscosity of host liquid enhanced. In addition, it is worthwhile to mention that the maximum magnitude of viscosity is attained at $$n=12.7$$ and $$\varphi $$ = 0.1 i.e. (0.0009). Figure 4 represents the impression of volume fraction parameter on axially directed velocity by fixing $$\alpha =-4,R=-1,M=1,Sc=1,Pr=6.2.$$ It is observed that the magnitude of the axial component of velocity near the lower wall decreases whereas contrary behavior is observed near the upper wall. The reason behind this fact is that we have prescribed a negative magnitude of velocity at the lower wall of the disk i.e. $$f$$ =  − 1 whereas the upper wall is prescribed with $$f$$ = 1. In addition, the increasing impact of nanoparticle volume fraction $$(\varphi )$$ is because increasing the magnitude $$(\varphi )$$ means the addition of more hybrid nanoparticles in the base fluid. So, by increasing $$(\varphi )$$ thermal conductivity of fluid increases, and thus as an outcome velocity of the fluid in the axial direction upsurges. Similar behavior in momentum distribution along radial direction against nanoparticle volume fraction $$(\varphi )$$ is divulged in Fig. 5. It is salient to notice that by increasing $$(\varphi 
)$$ the amount of metallic and non-metallic nanoparticles increases in the base fluid and considering $$(\varphi =0)$$ means that only base fluid is remaining. So, the purpose of considering nanoparticles is to enhance the velocity which is achieved and proved graphically from these sketches. Figure 6 shows the impact of Reynold number (R) on the axial velocity. After analyzing the graph, it is seen that the opposite behavior of the axial component of velocity against (R) is depicted. At the region near the lower wall velocity decreases whereas close to the upper wall region velocity distribution exhibits increasing aspects. The behavior of velocity at the lower wall is justified by the fact that we have considered permeability and fluid are moved out from the domain so the amount of fluid decays near the wall. Whereas, near upper wall-flow become stable and since increasing (R) magnitude of inertial forces increases thus the velocity distribution enhances. Figure 7 shows a deviation in radial component of velocity against magnetic parameter (M) and by restricting $$\alpha =5,{\varphi }_{1}={\varphi }_{2}=0.01,M=1,Sc=1,Pr=6.2$$. Declining behavior in radial component of velocity is measured against increasing magnitude of (M). Likewise, to previous figures, different aspects in velocity near the lower and upper walls are attained. Overall, the velocity of fluid depreciates against (M) because frictional forces are generated due to the intensification of Lorentz resistive forces which are applied in the orthogonal direction to retain the fluid in the laminar flow regime. Figure 8 displays the influence of magnetic parameter (M) on axially directed velocity distribution. Diminishing behavior in velocity against increasing magnitude of (M) is observed. It is due to the fact that the appliance of the magnetic field possesses the traits to resist the flow of particles and effect in reducing kinetic energy. It also produces resistive forces in the flow field which slows down the movement of particles.

**Table 2:** Deviation in-wall drag factor against $\varphi , R$, $\alpha$ $({\text{TiO}}_{2}{-}\text{Cu}/water$) (*HN*_*fd*_).

${\text{TiO}}_{2}{-}\text{Cu}/water$							${\left|{Nu}_{z}(5.7)\right|}_{\eta =-1}$
$\varphi$	$R$	$\alpha$	${\left|{C}_{f}\right|}_{\eta =-1}$	${\left|{Nu}_{z}(3)\right|}_{\eta =-1}$	${\left|{Nu}_{z}(3.7)\right|}_{\eta =-1}$	${\left|{Nu}_{z}(4.8)\right|}_{\eta =-1}$	
0.01	0.1	1	2.3728	0.1065	0.1104	0.1162	0.1208
0.02			2.3345	0.1170	0.1228	0.1317	0.1386
0.03			2.3023	0.1278	0.1356	0.1473	0.1563
0.04			2.2761	0.0870	0.1484	0.1692	0.1738
0.05	0	1	2.2412	0.1386	0.0956	0.1087	0.1189
	0.1		2.2761	0.2172	0.1484	0.1629	0.1738
	0.2		2.3141	0.3266	0.2254	0.2392	0.2495
	0.3		2.3557	0.4557	0.3266	0.3437	0.3509
0.05	0.1	0.3	2.2976	0.3307	0.4578	0.4607	0.4628
		0.5	2.7380	0.2361	0.3378	0.3476	0.3547
		0.7	2.5510	0.1386	0.2455	0.2589	0.2688
		1	2.2760	0.1695	0.1484	0.1629	0.1738

**Table 3:** Comparison of non-dimensionless physical variable effect against surface drag coefficient and convective heat transfer.

$\alpha$	$R$	$M$	${\varphi }_{1}$	${\varphi }_{2}$	${n}_{1}$	${n}_{2}$	$Pr$	${\left|{C}_{f}\right|}_{\eta =-1}$	${\left|{Nu}_{z}\right|}_{\eta =-1}$
0.1	0.01	1	0.01	0.01	3	3	6.2	3.1080	0.4302
0.2								$3.0262$	$0.3508$
0.3								$2.9437$	$0.2842$
0.4								$2.8623$	$0.2288$
	− 0.02							$3.0801$	$0.3362$
	− 0.03							$3.0591$	$0.2738$
	− 0.04							$3.0485$	$0.2464$
		− 1						$$\begin{array}{c}2.723\end{array}1$$	$0.4304$
		− 2						$$\begin{array}{c}2.5132\end{array}$$	$0.4305$
		− 3						$2.2893$	$0.4306$
			0.02					$3.0999$	$0.4322$
			0.03					$3.0922$	$0.4341$
			0.04					$3.0848$	$0.4360$
				0.02				$3.1031$	$0.4319$
				0.03				$3.0984$	$0.4336$
				0.04				$3.0939$	$0.4353$
					3.7			$3.1080$	$0.4302$
					4.8			$3.108$ 0	$0.4306$
					5.7			3.1080	$0.4311$
						3.7		3.1080	$0.4305$
						4.8		3.1080	$0.4308$
						5.7		3.1080	$0.4311$
							4.5	3.1080	$0.4485$
							5.2	$3.108$ 0	$0.4409$
							5.5	$3.108$ 0	$0.4376$

**Table 4:** Accuracy of missing initial conditions.

$\eta$	$f(-1)$	$f{^{\prime}}(-1)$	$f{^{\prime}}{^{\prime}}(-1)$
− 1	− 1	0	$$\begin{array}{c}2.868218\end{array}$$
$-0.9$	$-0.98600$	$$\begin{array}{c}0.276290\end{array}$$	$$\begin{array}{c}2.651363\end{array}$$
$-0.8$	$$\begin{array}{c}-0.945522\end{array}$$	$$\begin{array}{c}0.529198\end{array}$$	$$\begin{array}{c}2.402233\end{array}$$
$-0.7$	$$\begin{array}{c}-0.88103\end{array}$$	$$\begin{array}{c}0.755966\end{array}$$	$$\begin{array}{c}2.129942\end{array}$$
$-0.6$	$$\begin{array}{c}-0.795265\end{array}$$	$$\begin{array}{c}0.954662\end{array}$$	$$\begin{array}{c}1.841861\end{array}$$
$-0.5$	$$\begin{array}{c}-0.691083\end{array}$$	$$\begin{array}{c}1.124001\end{array}$$	$$\begin{array}{c}1.543588\end{array}$$
$-0.4$	$$\begin{array}{c}-0.571471\end{array}$$	$$\begin{array}{c}1.263175\end{array}$$	$$\begin{array}{c}1.239131\end{array}$$
$-0.3$	$$\begin{array}{c}-0.439470\end{array}$$	$$\begin{array}{c}1.371712\end{array}$$	$$\begin{array}{c}0.931185\end{array}$$
$-0.2$	$$\begin{array}{c}-0.298158\end{array}$$	$1.449353$	$$\begin{array}{c}0.621439\end{array}$$
$-0.1$	$$\begin{array}{c}-0.150633\end{array}$$	$$\begin{array}{c}1.495973\end{array}$$	$$\begin{array}{c}0.310870\end{array}$$
$0$	$$\begin{array}{c}1.554843\times {10}^{-10}\end{array}$$	$$\begin{array}{c}1.511517\end{array}$$	$$\begin{array}{c}1.291349\times {10}^{-9}\end{array}$$
0.1	$$\begin{array}{c}0.150633\end{array}$$	$$\begin{array}{c}1.495973\end{array}$$	$$\begin{array}{c}0.310870\end{array}$$
0.2	$$\begin{array}{c}0.298158\end{array}$$	$1.449353$	$$\begin{array}{c}0.621439\end{array}$$
0.3	$$\begin{array}{c}0.439470\end{array}$$	$$\begin{array}{c}1.371712\end{array}$$	$$\begin{array}{c}0.931185\end{array}$$
0.4	$$\begin{array}{c}0.571471\end{array}$$	$$\begin{array}{c}1.263175\end{array}$$	$$\begin{array}{c}1.239131\end{array}$$
0.5	$$\begin{array}{c}0.691083\end{array}$$	$$\begin{array}{c}1.124001\end{array}$$	$$\begin{array}{c}1.543588\end{array}$$
0.6	$0.795265$	$$\begin{array}{c}0.954662\end{array}$$	$$\begin{array}{c}1.841861\end{array}$$
0.7	$0.881037$	$0.755966$	$$\begin{array}{c}2.129942\end{array}$$
0.8	$$\begin{array}{c}0.945522\end{array}$$	$$\begin{array}{c}0.529198\end{array}$$	$$\begin{array}{c}2.402233\end{array}$$
0.9	$0.986004$	$$\begin{array}{c}0.529198\end{array}$$	$$\begin{array}{c}2.651363\end{array}$$
1	1	0	$$\begin{array}{c}2.868212\end{array}$$

**Table 5:** Validation of present work with published work.

$\alpha$	$f{^{\prime}}{^{\prime}}(-1)$, Kashif et al.^49^	Present work	${\theta }^{{\prime}}\left(-1\right)$, Kashif et al.^50^	Present work
− 5	3.6501	3.6505	7.6396	7.6399
− 3	2.9411	2.9416	3.9055	3.9950
− 1	2.3737	2.3736	2.0733	2.0365
0	2.1374	2.1375	1.5333	1.5336
1	1.9289	1.9299	1.1459	1.1461
3	1.5840	1.5845	0.6607	0.6617
5	1.3169	1.3260	0.3975	0.3985

## Conclusion

The current effort is conducted to adumbrate hybrid nanoliquid flow between porous surfaces. Two types of nanoparticles, (metallic and nonmetallic) have been used with shape, size factors. Mathematical structuring containing empirical relations for capitalized hybrid nanoparticles are attained in ODE’s after conversion from PDE’s Numerical solution is heeded for Tabular and graphical visualization of associated distributions. As far as the novelty of this work is concerned it is seen that hybridization of metallic and non-metallic nanoparticles in the base fluid has not yet been done so far. In addition, the flow of fluid between two coaxially rotating disks is considered very sparsely and even not with presently assumed physical aspects. So, the authors have hoped that this work will give new ideas and direction to researchers.

The main outcomes are itemized below:

- By specifying shape factor at *n* = 5.7 heat flux is high than other shape factors values.
- Noteworthy variations in thermophysical attributes by changing the morphology of the nanoparticles are observed.
- Magnetic field variable tends to enhance skin friction and Nusselt number along with the insertion of hybridized particles.
- The induction of hybrid nanoparticles raises thermal features more than ordinary nanoparticles.
- By incrementing volume fraction magnitude thermal distribution elevates whereas Reynolds number produces a reduction in mentioned profile.
- Elevation in Nusselt number and depreciation in skin friction coefficient is found against increasing magnitude of nanoparticle volume fraction.
- The positive trend in skin friction coefficient is manifested against the increasing magnitude of Reynold number.
- By increasing the size and shape of hybrid nanoparticles thermal conductivity and viscosity of base fluid increase.

## Nomenclature

${B}_{o}$: Uniform magnetic field [T]
${F}_{\eta }$: The dimensionless radial velocity profile
${C}_{f}$: The total skin friction coefficient
${\theta }_{\eta }$: Dimensionless temperature profile
${C}_{p}$: Specific heat at constant pressure
$\sigma$: Electrical conductivity [($\text{m}^{3}\text{A}^{2}$)/kg]
$k$: Time depended coefficient
$$\upsilon $$: Kinematic viscosity [$$\text{m}^{2}$$/s]
M: Magnetic parameter
$$\mu $$: Dynamic viscosity [$$\text{P}_\text{a}$$ s]
$${P}_{r}$$: Prandtl number
$$\rho $$: Density [kg/$$\text{m}^{3}]$$
r, z: Cylindrical coordinates system
${\rho C}_{P}$: Volumetric heat capacity [J/(m^3^ K)]
*R*: Reynolds number
T: ($${HN}_{fd}$$) temperature [K]
W: Mass or velocity component along z-axis
${A}_{1}$: Dimensionless parameter coefficient
Nu: Nusselt number
${{(\rho c}_{p})}_{hnf}$: Specific heat capacity
$${\rho }_{hnf}$$: Density for ($${HN}_{fd}$$)
${k}_{hnf}$: Thermal conductivity for (${HN}_{fd}$)
${\rho }_{s2}$: Density for second solid NP’s
${\rho }_{s1}$: Density for first solid NP’s
${k}_{s1}$: Thermal conductivity for a first solid fraction
${k}_{s2}$: Thermal conductivity for a second solid fraction
${k}_{mbf}$: Thermal conductivity for shape base fluid
P: Pressure
${k}_{bf}$: Thermal conductivity for base fluid
${\mu }_{eff}$: Viscosity for effect
${\mu }_{bf}$: Viscosity base fluid
$dp$: Dimeter of particles
${\upsilon }_{hnf}$: Kinematics viscosity for (${HN}_{fd}$)
$${Nu}_{z}$$: Nusselt number

## Subscripts

$${B}_{fd}$$: Base fluid
$${N}_{fd}$$: Nanofluid
$${HN}_{fd}$$: Hybrid nanofluid
1: First nanoparticle ($${\text{TiO}}_{2}$$)
2: Second nanoparticle (Cu)

## Greek symbols

$$\alpha $$: Thermal diffusivity [m^2^/s]
$\eta$: Independent similarity variable
$\varphi$: Equivalent nanoparticles volume fraction
${\varphi }_{1}$: Equivalent first nanoparticles volume fraction
${\varphi }_{2}$: Equivalent first nanoparticles volume fraction
